# Supplementary material for: Multi-assay approach shows species-associated personality patterns in two socially distinct gerbil species
Source: PLoS One. 2024 Apr 16;19(4):e0296214. doi: 10.1371/journal.pone.0296214 (PMC11020386; doi:10.1371/journal.pone.0296214)
Supplement: S2 File — (DOCX) [file pone.0296214.s002.docx]

**Multi-assay approach shows species-associated personality patterns in two socially distinct gerbil species**

Andrey V. Tchabovsky^1*^, Elena N. Surkova^1^, Ludmila E. Savinetskaya^1^

**Supporting information**

**Codes**

**#(1)Phenotypic correlations**

#Pairwise correlations between behavioural measurements

#using bivariate Bayesian generalized linear mixed-effect models

#with Markov chain Monte Carlo (MCMC) techniques

#implemented in the R package MCMCglmm

#with animal ID included as random factor (Tables 2 and 6)

library(MCMCglmm)

prior2 <- list(G = list(G1 = list(V = diag(2), nu = 0.004)),

R = list(V = diag(2), nu = 0.002))

#Data: pers_lab.csv; S1_File.csv in Supporting information files

#should be renamed as pers_lab.csv

#Repeated measures

#Random = animal ID

#95%CI by HPD

pers_lab <- read.csv("D:/Work/R/data/pers_lab.csv",header=TRUE)

pers_lab$trial<- as.factor(pers_lab$trial)

pers_lab$species<- as.factor(pers_lab$species)

pers_lab$animal<- as.factor(pers_lab$animal)

pers_lab$sex<- as.factor(pers_lab$sex)

#**M.meridianus**

pers_lab_mm <- subset(pers_lab, species == "mm")

#**Table 2 in the main text:**

#Correlations between the same measurements of boldness

#across three tests (contexts) derived from

#the bivariate MCMCglmm models

#with animal ID included as random factor.

#HO_ST:HO_EP, head out in DL/S/NO vs head out in DL/EP

model2.1 <- MCMCglmm(cbind(ho_st, ho_ep) ~ trait - 1,

random = ~ us(trait):animal, rcov = ~ us(trait):units,

family = c("gaussian", "gaussian"),

data = pers_lab_mm, nitt = 650000, thin = 1000, burnin = 150000,

prior = prior2, verbose = FALSE)

autocorr.diag(model2.1$VCV)

plot(model2.1$Sol)

#Phenotypic (Within + Among) Correlation

genetic.correlation2.3 <- (model2.1$VCV[,

"traitho_st:traitho_ep.animal"] + model2.1$VCV[,

"traitho_st:traitho_ep.units"]) / sqrt((model2.1$VCV[,

"traitho_st:traitho_st.animal"] + model2.1$VCV[,

"traitho_st:traitho_st.units"]) * (model2.1$VCV[,

"traitho_ep:traitho_ep.animal"] + model2.1$VCV[,

"traitho_ep:traitho_ep.units"]))

posterior.mode(genetic.correlation2.3)

HPDinterval(genetic.correlation2.3, 0.95)

#HO_ST:HO_STR, head out in DL/S/NO vs head out in DL/STR

model2.1 <- MCMCglmm(cbind(ho_st, ho_str) ~ trait - 1,

random = ~ us(trait):animal,

rcov = ~ us(trait):units,

family = c("gaussian", "gaussian"),

data = pers_lab_mm, nitt = 650000, thin = 1000, burnin = 150000,

prior = prior2, verbose = FALSE)

plot(model2.1$Sol)

autocorr.diag(model2.1$VCV)

#Phenotypic (Within + Among) Correlation

genetic.correlation2.3 <- (model2.1$VCV[,

"traitho_st:traitho_str.animal"] + model2.1$VCV[,

"traitho_st:traitho_str.units"]) / sqrt((model2.1$VCV[,

"traitho_st:traitho_st.animal"] + model2.1$VCV[,

"traitho_st:traitho_st.units"]) * (model2.1$VCV[,

"traitho_str:traitho_str.animal"] + model2.1$VCV[,

"traitho_str:traitho_str.units"]))

posterior.mode(genetic.correlation2.3)

HPDinterval(genetic.correlation2.3, 0.95)

#HO_EP:HO_STR, head out in DL/EP vs head out in DL/STR

model2.1 <- MCMCglmm(cbind(ho_ep, ho_str) ~ trait - 1,

random = ~ us(trait):animal,

rcov = ~ us(trait):units,

family = c("gaussian", "gaussian"),

data = pers_lab_mm, nitt = 650000, thin = 1000, burnin = 150000,

prior = prior2, verbose = FALSE)

plot(model2.1$Sol)

autocorr.diag(model2.1$VCV)

#Phenotypic (Within + Among) Correlation

genetic.correlation2.3 <- (model2.1$VCV[,

"traitho_ep:traitho_str.animal"] + model2.1$VCV[,

"traitho_ep:traitho_str.units"]) / sqrt((model2.1$VCV[,

"traitho_ep:traitho_ep.animal"] + model2.1$VCV[,

"traitho_ep:traitho_ep.units"]) * (model2.1$VCV[,

"traitho_str:traitho_str.animal"] + model2.1$VCV[,

"traitho_str:traitho_str.units"]))

posterior.mode(genetic.correlation2.3)

HPDinterval(genetic.correlation2.3, 0.95)

#BO_EP:BO_STR, body out in DL/EP vs body out in DL/STR

model2.1 <- MCMCglmm(cbind(bo_ep, bo_str) ~ trait - 1,

random = ~ us(trait):animal,

rcov = ~ us(trait):units,

family = c("gaussian", "gaussian"),

data = pers_lab_mm, nitt = 650000, thin = 1000, burnin = 150000,

prior = prior2, verbose = FALSE)

plot(model2.1$Sol)

autocorr.diag(model2.1$VCV)

#Phenotypic (Within + Among) Correlation

genetic.correlation2.3 <- (model2.1$VCV[,

"traitbo_ep:traitbo_str.animal"] + model2.1$VCV[,

"traitbo_ep:traitbo_str.units"]) / sqrt((model2.1$VCV[,

"traitbo_ep:traitbo_ep.animal"] + model2.1$VCV[,

"traitbo_ep:traitbo_ep.units"]) * (model2.1$VCV[,

"traitbo_str:traitbo_str.animal"] + model2.1$VCV[,

"traitbo_str:traitbo_str.units"]))

posterior.mode(genetic.correlation2.3)

HPDinterval(genetic.correlation2.3, 0.95)

#BO_EP:BO_ST, body out in DL/EP vs body out in DL/S/NO

model2.1 <- MCMCglmm(cbind(bo_ep, bo_st) ~ trait - 1,

random = ~ us(trait):animal,

rcov = ~ us(trait):units,

family = c("gaussian", "gaussian"),

data = pers_lab_mm, nitt = 650000, thin = 1000, burnin = 150000,

prior = prior2, verbose = FALSE)

plot(model2.1$Sol)

autocorr.diag(model2.1$VCV)

#Phenotypic (Within + Among) Correlation

genetic.correlation2.3 <- (model2.1$VCV[,

"traitbo_ep:traitbo_st.animal"] + model2.1$VCV[,

"traitbo_ep:traitbo_st.units"]) / sqrt((model2.1$VCV[,

"traitbo_ep:traitbo_ep.animal"] + model2.1$VCV[,

"traitbo_ep:traitbo_ep.units"]) * (model2.1$VCV[,

"traitbo_st:traitbo_st.animal"] + model2.1$VCV[,

"traitbo_st:traitbo_st.units"]))

posterior.mode(genetic.correlation2.3)

HPDinterval(genetic.correlation2.3, 0.95)

#BO_STR:BO_ST, body out in DL/STR vs body out in DL/S/NO

model2.1 <- MCMCglmm(cbind(bo_str, bo_st) ~ trait - 1,

random = ~ us(trait):animal,

rcov = ~ us(trait):units,

family = c("gaussian", "gaussian"),

data = pers_lab_mm, nitt = 650000, thin = 1000, burnin = 150000,

prior = prior2, verbose = FALSE)

plot(model2.1$Sol)

autocorr.diag(model2.1$VCV)

#Phenotypic-(withn - + among variation) Correlation

genetic.correlation2.3 <- (model2.1$VCV[,

"traitbo_str:traitbo_st.animal"] + model2.1$VCV[,

"traitbo_str:traitbo_st.units"]) / sqrt((model2.1$VCV[,

"traitbo_str:traitbo_str.animal"] + model2.1$VCV[,

"traitbo_str:traitbo_str.units"]) * (model2.1$VCV[,

"traitbo_st:traitbo_st.animal"] + model2.1$VCV[,

"traitbo_st:traitbo_st.units"]))

posterior.mode(genetic.correlation2.3)

HPDinterval(genetic.correlation2.3, 0.95)

#**Table 6 in the main text:**

#Phenotypic correlations between different behaviours

#derived from the bivariate MCMCglmm models

#with animal ID included as random factor

#IMMOB:CD, immobility in the bag vs climb down in DL/EP

model2.1 <- MCMCglmm(cbind(immob, cd) ~ trait - 1,

random = ~ us(trait):animal,

rcov = ~ us(trait):units,

family = c("gaussian", "gaussian"),

data = pers_lab_mm, nitt = 650000, thin = 1000, burnin = 150000,

prior = prior2, verbose = FALSE)

plot(model2.1$Sol)

autocorr.diag(model2.1$VCV)

#Phenotypic-(within- + among variation) Correlation

genetic.correlation2.3 <- (model2.1$VCV[,

"traitimmob:traitcd.animal"] + model2.1$VCV[,

"traitimmob:traitcd.units"]) / sqrt((model2.1$VCV[,

"traitimmob:traitimmob.animal"] + model2.1$VCV[,

"traitimmob:traitimmob.units"]) * (model2.1$VCV[,

"traitcd:traitcd.animal"] + model2.1$VCV[,

"traitcd:traitcd.units"]))

posterior.mode(genetic.correlation2.3)

HPDinterval(genetic.correlation2.3, 0.95)

#IMMOB:CS, immobility in the bag vs contact stranger in DL/STR

model2.1 <- MCMCglmm(cbind(immob, cs) ~ trait - 1,

random = ~ us(trait):animal,

rcov = ~ us(trait):units,

family = c("gaussian", "gaussian"),

data = pers_lab_mm, nitt = 650000, thin = 1000, burnin = 150000,

prior = prior2, verbose = FALSE)

plot(model2.1$Sol)

autocorr.diag(model2.1$VCV)

#Phenotypic-(within- + among variation) Correlation

genetic.correlation2.3 <- (model2.1$VCV[,

"traitimmob:traitcs.animal"] + model2.1$VCV[,

"traitimmob:traitcs.units"]) / sqrt((model2.1$VCV[,

"traitimmob:traitimmob.animal"] + model2.1$VCV[,

"traitimmob:traitimmob.units"]) * (model2.1$VCV[,

"traitcs:traitcs.animal"] + model2.1$VCV[,

"traitcs:traitcs.units"]))

posterior.mode(genetic.correlation2.3)

HPDinterval(genetic.correlation2.3, 0.95)

#IMMOB:CO, immobility in the bag vs contact object in DL/S/NO

model2.1 <- MCMCglmm(cbind(immob, co) ~ trait - 1,

random = ~ us(trait):animal,

rcov = ~ us(trait):units,

family = c("gaussian", "gaussian"),

data = pers_lab_mm, nitt = 650000, thin = 1000, burnin = 150000,

prior = prior2, verbose = FALSE)

plot(model2.1$Sol)

autocorr.diag(model2.1$VCV)

#Phenotypic-(within- + among variation) Correlation

genetic.correlation2.3 <- (model2.1$VCV[,

"traitimmob:traitco.animal"] + model2.1$VCV[,

"traitimmob:traitco.units"]) / sqrt((model2.1$VCV[,

"traitimmob:traitimmob.animal"] + model2.1$VCV[,

"traitimmob:traitimmob.units"]) * (model2.1$VCV[,

"traitco:traitco.animal"] + model2.1$VCV[,

"traitco:traitco.units"]))

posterior.mode(genetic.correlation2.3)

HPDinterval(genetic.correlation2.3, 0.95)

#IMMOB:BO_ST, immobility in the bag vs body out in DL/S/NO

model2.1 <- MCMCglmm(cbind(immob, bo_st) ~ trait - 1,

random = ~ us(trait):animal,

rcov = ~ us(trait):units,

family = c("gaussian", "gaussian"),

data = pers_lab_mm, nitt = 650000, thin = 1000, burnin = 150000,

prior = prior2, verbose = FALSE)

plot(model2.1$Sol)

autocorr.diag(model2.1$VCV)

#Phenotypic-(within- + among variation) Correlation

genetic.correlation2.3 <- (model2.1$VCV[,

"traitimmob:traitbo_st.animal"] + model2.1$VCV[,

"traitimmob:traitbo_st.units"]) / sqrt((model2.1$VCV[,

"traitimmob:traitimmob.animal"] + model2.1$VCV[,

"traitimmob:traitimmob.units"]) * (model2.1$VCV[,

"traitbo_st:traitbo_st.animal"] + model2.1$VCV[,

"traitbo_st:traitbo_st.units"]))

posterior.mode(genetic.correlation2.3)

HPDinterval(genetic.correlation2.3, 0.95)

#BO_ST:CS, body out in DL/S/NO vs contact stranger in DL/STR

model2.1 <- MCMCglmm(cbind(bo_st, cs) ~ trait - 1,

random = ~ us(trait):animal,

rcov = ~ us(trait):units,

family = c("gaussian", "gaussian"),

data = pers_lab_mm, nitt = 650000, thin = 1000, burnin = 150000,

prior = prior2, verbose = FALSE)

plot(model2.1$Sol)

autocorr.diag(model2.1$VCV)

#Phenotypic-(within- + among variation) Correlation

genetic.correlation2.3 <- (model2.1$VCV[,

"traitbo_st:traitcs.animal"] + model2.1$VCV[,

"traitbo_st:traitcs.units"]) / sqrt((model2.1$VCV[,

"traitbo_st:traitbo_st.animal"] + model2.1$VCV[,

"traitbo_st:traitbo_st.units"]) * (model2.1$VCV[,

"traitcs:traitcs.animal"] + model2.1$VCV[,

"traitcs:traitcs.units"]))

posterior.mode(genetic.correlation2.3)

HPDinterval(genetic.correlation2.3, 0.95)

#BO_ST:CD, body out in DL/S/NO vs climb down in DL/EP

model2.1 <- MCMCglmm(cbind(bo_st, cd) ~ trait - 1,

random = ~ us(trait):animal,

rcov = ~ us(trait):units,

family = c("gaussian", "gaussian"),

data = pers_lab_mm, nitt = 650000, thin = 1000, burnin = 150000,

prior = prior2, verbose = FALSE)

plot(model2.1$Sol)

autocorr.diag(model2.1$VCV)

#Phenotypic-(within- + among variation) Correlation

genetic.correlation2.3 <- (model2.1$VCV[,

"traitbo_st:traitcd.animal"] + model2.1$VCV[,

"traitbo_st:traitcd.units"]) / sqrt((model2.1$VCV[,

"traitbo_st:traitbo_st.animal"] + model2.1$VCV[,

"traitbo_st:traitbo_st.units"]) * (model2.1$VCV[,

"traitcd:traitcd.animal"] + model2.1$VCV[,

"traitcd:traitcd.units"]))

posterior.mode(genetic.correlation2.3)

HPDinterval(genetic.correlation2.3, 0.95)

#BO_ST:CO, body out in DL/S/NO vs contact object in DL/S/NO

model2.1 <- MCMCglmm(cbind(bo_st, co) ~ trait - 1,

random = ~ us(trait):animal,

rcov = ~ us(trait):units,

family = c("gaussian", "gaussian"),

data = pers_lab_mm, nitt = 650000, thin = 1000, burnin = 150000,

prior = prior2, verbose = FALSE)

plot(model2.1$Sol)

autocorr.diag(model2.1$VCV)

#Phenotypic-(within- + among variation) Correlation

genetic.correlation2.3 <- (model2.1$VCV[,

"traitbo_st:traitco.animal"] + model2.1$VCV[,

"traitbo_st:traitco.units"]) / sqrt((model2.1$VCV[,

"traitbo_st:traitbo_st.animal"] + model2.1$VCV[,

"traitbo_st:traitbo_st.units"]) * (model2.1$VCV[,

"traitco:traitco.animal"] + model2.1$VCV[,

"traitco:traitco.units"]))

posterior.mode(genetic.correlation2.3)

HPDinterval(genetic.correlation2.3, 0.95)

#CO:CD, contact object in DL/S/NO vs climb down in DL/EP

model2.1 <- MCMCglmm(cbind(cd, co) ~ trait - 1,

random = ~ us(trait):animal,

rcov = ~ us(trait):units,

family = c("gaussian", "gaussian"),

data = pers_lab_mm, nitt = 650000, thin = 1000, burnin = 150000,

prior = prior2, verbose = FALSE)

plot(model2.1$Sol)

autocorr.diag(model2.1$VCV)

#Phenotypic-(within- + among variation) Correlation

genetic.correlation2.3 <- (model2.1$VCV[,

"traitcd:traitco.animal"] + model2.1$VCV[,

"traitcd:traitco.units"]) / sqrt((model2.1$VCV[,

"traitcd:traitcd.animal"] + model2.1$VCV[,

"traitcd:traitcd.units"]) * (model2.1$VCV[,

"traitco:traitco.animal"] + model2.1$VCV[,

"traitco:traitco.units"]))

posterior.mode(genetic.correlation2.3)

HPDinterval(genetic.correlation2.3, 0.95)

#CO:CS, contact object in DL/S/NO vs contact stranger in DL/STR

model2.1 <- MCMCglmm(cbind(cs, co) ~ trait - 1,

random = ~ us(trait):animal,

rcov = ~ us(trait):units,

family = c("gaussian", "gaussian"),

data = pers_lab_mm, nitt = 650000, thin = 1000, burnin = 150000,

prior = prior2, verbose = FALSE)

plot(model2.1$Sol)

autocorr.diag(model2.1$VCV)

#Phenotypic-(within- + among variation) Correlation

genetic.correlation2.3 <- (model2.1$VCV[,

"traitcs:traitco.animal"] + model2.1$VCV[,

"traitcs:traitco.units"]) / sqrt((model2.1$VCV[,

"traitcs:traitcs.animal"] + model2.1$VCV[,

"traitcs:traitcs.units"]) * (model2.1$VCV[,

"traitco:traitco.animal"] + model2.1$VCV[,

"traitco:traitco.units"]))

posterior.mode(genetic.correlation2.3)

HPDinterval(genetic.correlation2.3, 0.95)

#CD:CS, climb down in DL/EP vs contact stranger in DL/STR

model2.1 <- MCMCglmm(cbind(cs, cd) ~ trait - 1,

random = ~ us(trait):animal,

rcov = ~ us(trait):units,

family = c("gaussian", "gaussian"),

data = pers_lab_mm, nitt = 650000, thin = 1000, burnin = 150000,

prior = prior2, verbose = FALSE)

plot(model2.1$Sol)

autocorr.diag(model2.1$VCV)

#Phenotypic-(within- + among variation) Correlation

genetic.correlation2.3 <- (model2.1$VCV[,

"traitcs:traitcd.animal"] + model2.1$VCV[,

"traitcs:traitcd.units"]) / sqrt((model2.1$VCV[,

"traitcs:traitcs.animal"] + model2.1$VCV[,

"traitcs:traitcs.units"]) * (model2.1$VCV[,

"traitcd:traitcd.animal"] + model2.1$VCV[,

"traitcd:traitcd.units"]))

posterior.mode(genetic.correlation2.3)

HPDinterval(genetic.correlation2.3, 0.95)

#**M.unguiculatus**

#Data: pers_lab.csv

#Repeated measures

#Random = animal ID

#95%CI by HPD

pers_lab_mu <- subset(pers_lab, species == "mu")

#**Table 2 in the main text**:

#Correlations between the same measurements of boldness

#across three tests (contexts) derived from

#the bivariate MCMCglmm models

#with animal ID included as random factor. )

#HO_ST:HO_EP, head out in DL/S/NO vs head out in DL/EP

model2.1 <- MCMCglmm(cbind(ho_st, ho_ep) ~ trait - 1,

random = ~ us(trait):animal, rcov = ~ us(trait):units,

family = c("gaussian", "gaussian"),

data = pers_lab_mu, nitt = 650000, thin = 1000, burnin = 150000,

prior = prior2, verbose = FALSE)

autocorr.diag(model2.1$VCV)

plot(model2.1$Sol)

#Phenotypic (Within + Among) Correlation

genetic.correlation2.3 <- (model2.1$VCV[,

"traitho_st:traitho_ep.animal"] + model2.1$VCV[,

"traitho_st:traitho_ep.units"]) / sqrt((model2.1$VCV[,

"traitho_st:traitho_st.animal"] + model2.1$VCV[,

"traitho_st:traitho_st.units"]) * (model2.1$VCV[,

"traitho_ep:traitho_ep.animal"] + model2.1$VCV[,

"traitho_ep:traitho_ep.units"]))

posterior.mode(genetic.correlation2.3)

HPDinterval(genetic.correlation2.3, 0.95)

#HO_ST:HO_STR, head out in DL/S/NO vs head out in DL/STR

model2.1 <- MCMCglmm(cbind(ho_st, ho_str) ~ trait - 1,

random = ~ us(trait):animal,

rcov = ~ us(trait):units,

family = c("gaussian", "gaussian"),

data = pers_lab_mu, nitt = 650000, thin = 1000, burnin = 150000,

prior = prior2, verbose = FALSE)

plot(model2.1$Sol)

autocorr.diag(model2.1$VCV)

#Phenotypic (Within + Among) Correlation

genetic.correlation2.3 <- (model2.1$VCV[,

"traitho_st:traitho_str.animal"] + model2.1$VCV[,

"traitho_st:traitho_str.units"]) / sqrt((model2.1$VCV[,

"traitho_st:traitho_st.animal"] + model2.1$VCV[,

"traitho_st:traitho_st.units"]) * (model2.1$VCV[,

"traitho_str:traitho_str.animal"] + model2.1$VCV[,

"traitho_str:traitho_str.units"]))

posterior.mode(genetic.correlation2.3)

HPDinterval(genetic.correlation2.3, 0.95)

#HO_EP:HO_STR, head out in DL/EP vs head out in DL/STR

model2.1 <- MCMCglmm(cbind(ho_ep, ho_str) ~ trait - 1,

random = ~ us(trait):animal,

rcov = ~ us(trait):units,

family = c("gaussian", "gaussian"),

data = pers_lab_mu, nitt = 650000, thin = 1000, burnin = 150000,

prior = prior2, verbose = FALSE)

plot(model2.1$Sol)

autocorr.diag(model2.1$VCV)

#Phenotypic (Within + Among) Correlation

genetic.correlation2.3 <- (model2.1$VCV[,

"traitho_ep:traitho_str.animal"] + model2.1$VCV[,

"traitho_ep:traitho_str.units"]) / sqrt((model2.1$VCV[,

"traitho_ep:traitho_ep.animal"] + model2.1$VCV[,

"traitho_ep:traitho_ep.units"]) * (model2.1$VCV[,

"traitho_str:traitho_str.animal"] + model2.1$VCV[,

"traitho_str:traitho_str.units"]))

posterior.mode(genetic.correlation2.3)

HPDinterval(genetic.correlation2.3, 0.95)

#BO_EP:BO_STR, body out in DL/EP vs body out in DL/STR

model2.1 <- MCMCglmm(cbind(bo_ep, bo_str) ~ trait - 1,

random = ~ us(trait):animal,

rcov = ~ us(trait):units,

family = c("gaussian", "gaussian"),

data = pers_lab_mu, nitt = 650000, thin = 1000, burnin = 150000,

prior = prior2, verbose = FALSE)

plot(model2.1$Sol)

autocorr.diag(model2.1$VCV)

#Phenotypic (Within + Among) Correlation

genetic.correlation2.3 <- (model2.1$VCV[,

"traitbo_ep:traitbo_str.animal"] + model2.1$VCV[,

"traitbo_ep:traitbo_str.units"]) / sqrt((model2.1$VCV[,

"traitbo_ep:traitbo_ep.animal"] + model2.1$VCV[,

"traitbo_ep:traitbo_ep.units"]) * (model2.1$VCV[,

"traitbo_str:traitbo_str.animal"] + model2.1$VCV[,

"traitbo_str:traitbo_str.units"]))

posterior.mode(genetic.correlation2.3)

HPDinterval(genetic.correlation2.3, 0.95)

#BO_EP:BO_ST, body out in DL/EP vs body out in DL/S/NO

model2.1 <- MCMCglmm(cbind(bo_ep, bo_st) ~ trait - 1,

random = ~ us(trait):animal,

rcov = ~ us(trait):units,

family = c("gaussian", "gaussian"),

data = pers_lab_mu, nitt = 650000, thin = 1000, burnin = 150000,

prior = prior2, verbose = FALSE)

plot(model2.1$Sol)

autocorr.diag(model2.1$VCV)

#Phenotypic (Within + Among) Correlation

genetic.correlation2.3 <- (model2.1$VCV[,

"traitbo_ep:traitbo_st.animal"] + model2.1$VCV[,

"traitbo_ep:traitbo_st.units"]) / sqrt((model2.1$VCV[,

"traitbo_ep:traitbo_ep.animal"] + model2.1$VCV[,

"traitbo_ep:traitbo_ep.units"]) * (model2.1$VCV[,

"traitbo_st:traitbo_st.animal"] + model2.1$VCV[,

"traitbo_st:traitbo_st.units"]))

posterior.mode(genetic.correlation2.3)

HPDinterval(genetic.correlation2.3, 0.95)

#BO_STR:BO_ST, body out in DL/STR vs body out in DL/S/NO

model2.1 <- MCMCglmm(cbind(bo_str, bo_st) ~ trait - 1,

random = ~ us(trait):animal,

rcov = ~ us(trait):units,

family = c("gaussian", "gaussian"),

data = pers_lab_mu, nitt = 650000, thin = 1000, burnin = 150000,

prior = prior2, verbose = FALSE)

plot(model2.1$Sol)

autocorr.diag(model2.1$VCV)

#Phenotypic-(withn - + among variation) Correlation

genetic.correlation2.3 <- (model2.1$VCV[,

"traitbo_str:traitbo_st.animal"] + model2.1$VCV[,

"traitbo_str:traitbo_st.units"]) / sqrt((model2.1$VCV[,

"traitbo_str:traitbo_str.animal"] + model2.1$VCV[,

"traitbo_str:traitbo_str.units"]) * (model2.1$VCV[,

"traitbo_st:traitbo_st.animal"] + model2.1$VCV[,

"traitbo_st:traitbo_st.units"]))

posterior.mode(genetic.correlation2.3)

HPDinterval(genetic.correlation2.3, 0.95)

#**Table 6 in the main text**:

#Phenotypic correlations between different behaviours

#derived from the bivariate MCMCglmm models

#with animal ID included as random factor

#IMMOB:CD, immobility in the bag vs climb down in DL/EP

model2.1 <- MCMCglmm(cbind(immob, cd) ~ trait - 1,

random = ~ us(trait):animal,

rcov = ~ us(trait):units,

family = c("gaussian", "gaussian"),

data = pers_lab_mu, nitt = 650000, thin = 1000, burnin = 150000,

prior = prior2, verbose = FALSE)

plot(model2.1$Sol)

autocorr.diag(model2.1$VCV)

#Phenotypic-(within- + among variation) Correlation

genetic.correlation2.3 <- (model2.1$VCV[,

"traitimmob:traitcd.animal"] + model2.1$VCV[,

"traitimmob:traitcd.units"]) / sqrt((model2.1$VCV[,

"traitimmob:traitimmob.animal"] + model2.1$VCV[,

"traitimmob:traitimmob.units"]) * (model2.1$VCV[,

"traitcd:traitcd.animal"] + model2.1$VCV[,

"traitcd:traitcd.units"]))

posterior.mode(genetic.correlation2.3)

HPDinterval(genetic.correlation2.3, 0.95)

#IMMOB:CS, immobility in the bag vs contact stranger in DL/STR

model2.1 <- MCMCglmm(cbind(immob, cs) ~ trait - 1,

random = ~ us(trait):animal,

rcov = ~ us(trait):units,

family = c("gaussian", "gaussian"),

data = pers_lab_mu, nitt = 650000, thin = 1000, burnin = 150000,

prior = prior2, verbose = FALSE)

plot(model2.1$Sol)

autocorr.diag(model2.1$VCV)

#Phenotypic-(within- + among variation) Correlation

genetic.correlation2.3 <- (model2.1$VCV[,

"traitimmob:traitcs.animal"] + model2.1$VCV[,

"traitimmob:traitcs.units"]) / sqrt((model2.1$VCV[,

"traitimmob:traitimmob.animal"] + model2.1$VCV[,

"traitimmob:traitimmob.units"]) * (model2.1$VCV[,

"traitcs:traitcs.animal"] + model2.1$VCV[,

"traitcs:traitcs.units"]))

posterior.mode(genetic.correlation2.3)

HPDinterval(genetic.correlation2.3, 0.95)

#IMMOB:CO, immobility in the bag vs contact object in DL/S/NO

model2.1 <- MCMCglmm(cbind(immob, co) ~ trait - 1,

random = ~ us(trait):animal,

rcov = ~ us(trait):units,

family = c("gaussian", "gaussian"),

data = pers_lab_mu, nitt = 650000, thin = 1000, burnin = 150000,

prior = prior2, verbose = FALSE)

plot(model2.1$Sol)

autocorr.diag(model2.1$VCV)

#Phenotypic-(within- + among variation) Correlation

genetic.correlation2.3 <- (model2.1$VCV[,

"traitimmob:traitco.animal"] + model2.1$VCV[,

"traitimmob:traitco.units"]) / sqrt((model2.1$VCV[,

"traitimmob:traitimmob.animal"] + model2.1$VCV[,

"traitimmob:traitimmob.units"]) * (model2.1$VCV[,

"traitco:traitco.animal"] + model2.1$VCV[,

"traitco:traitco.units"]))

posterior.mode(genetic.correlation2.3)

HPDinterval(genetic.correlation2.3, 0.95)

#IMMOB:BO_ST, immobility in the bag vs body out in DL/S/NO

model2.1 <- MCMCglmm(cbind(immob, bo_st) ~ trait - 1,

random = ~ us(trait):animal,

rcov = ~ us(trait):units,

family = c("gaussian", "gaussian"),

data = pers_lab_mu, nitt = 650000, thin = 1000, burnin = 150000,

prior = prior2, verbose = FALSE)

plot(model2.1$Sol)

autocorr.diag(model2.1$VCV)

#Phenotypic-(within- + among variation) Correlation

genetic.correlation2.3 <- (model2.1$VCV[,

"traitimmob:traitbo_st.animal"] + model2.1$VCV[,

"traitimmob:traitbo_st.units"]) / sqrt((model2.1$VCV[,

"traitimmob:traitimmob.animal"] + model2.1$VCV[,

"traitimmob:traitimmob.units"]) * (model2.1$VCV[,

"traitbo_st:traitbo_st.animal"] + model2.1$VCV[,

"traitbo_st:traitbo_st.units"]))

posterior.mode(genetic.correlation2.3)

HPDinterval(genetic.correlation2.3, 0.95)

#BO_ST:CS, body out in DL/S/NO vs contact stranger in DL/STR

model2.1 <- MCMCglmm(cbind(bo_st, cs) ~ trait - 1,

random = ~ us(trait):animal,

rcov = ~ us(trait):units,

family = c("gaussian", "gaussian"),

data = pers_lab_mu, nitt = 650000, thin = 1000, burnin = 150000,

prior = prior2, verbose = FALSE)

plot(model2.1$Sol)

autocorr.diag(model2.1$VCV)

#Phenotypic-(within- + among variation) Correlation

genetic.correlation2.3 <- (model2.1$VCV[,

"traitbo_st:traitcs.animal"] + model2.1$VCV[,

"traitbo_st:traitcs.units"]) / sqrt((model2.1$VCV[,

"traitbo_st:traitbo_st.animal"] + model2.1$VCV[,

"traitbo_st:traitbo_st.units"]) * (model2.1$VCV[,

"traitcs:traitcs.animal"] + model2.1$VCV[,

"traitcs:traitcs.units"]))

posterior.mode(genetic.correlation2.3)

HPDinterval(genetic.correlation2.3, 0.95)

#BO_ST:CD, body out in DL/S/NO vs climb down in DL/EP

model2.1 <- MCMCglmm(cbind(bo_st, cd) ~ trait - 1,

random = ~ us(trait):animal,

rcov = ~ us(trait):units,

family = c("gaussian", "gaussian"),

data = pers_lab_mu, nitt = 650000, thin = 1000, burnin = 150000,

prior = prior2, verbose = FALSE)

plot(model2.1$Sol)

autocorr.diag(model2.1$VCV)

#Phenotypic-(within- + among variation) Correlation

genetic.correlation2.3 <- (model2.1$VCV[,

"traitbo_st:traitcd.animal"] + model2.1$VCV[,

"traitbo_st:traitcd.units"]) / sqrt((model2.1$VCV[,

"traitbo_st:traitbo_st.animal"] + model2.1$VCV[,

"traitbo_st:traitbo_st.units"]) * (model2.1$VCV[,

"traitcd:traitcd.animal"] + model2.1$VCV[,

"traitcd:traitcd.units"]))

posterior.mode(genetic.correlation2.3)

HPDinterval(genetic.correlation2.3, 0.95)

#BO_ST:CO, body out in DL/S/NO vs contact object in DL/S/NO

model2.1 <- MCMCglmm(cbind(bo_st, co) ~ trait - 1,

random = ~ us(trait):animal,

rcov = ~ us(trait):units,

family = c("gaussian", "gaussian"),

data = pers_lab_mu, nitt = 650000, thin = 1000, burnin = 150000,

prior = prior2, verbose = FALSE)

plot(model2.1$Sol)

autocorr.diag(model2.1$VCV)

#Phenotypic-(within- + among variation) Correlation

genetic.correlation2.3 <- (model2.1$VCV[,

"traitbo_st:traitco.animal"] + model2.1$VCV[,

"traitbo_st:traitco.units"]) / sqrt((model2.1$VCV[,

"traitbo_st:traitbo_st.animal"] + model2.1$VCV[,

"traitbo_st:traitbo_st.units"]) * (model2.1$VCV[,

"traitco:traitco.animal"] + model2.1$VCV[,

"traitco:traitco.units"]))

posterior.mode(genetic.correlation2.3)

HPDinterval(genetic.correlation2.3, 0.95)

#CO:CD, contact object in DL/S/NO vs climb down in DL/EP

model2.1 <- MCMCglmm(cbind(cd, co) ~ trait - 1,

random = ~ us(trait):animal,

rcov = ~ us(trait):units,

family = c("gaussian", "gaussian"),

data = pers_lab_mu, nitt = 650000, thin = 1000, burnin = 150000,

prior = prior2, verbose = FALSE)

plot(model2.1$Sol)

autocorr.diag(model2.1$VCV)

#Phenotypic-(within- + among variation) Correlation

genetic.correlation2.3 <- (model2.1$VCV[,

"traitcd:traitco.animal"] + model2.1$VCV[,

"traitcd:traitco.units"]) / sqrt((model2.1$VCV[,

"traitcd:traitcd.animal"] + model2.1$VCV[,

"traitcd:traitcd.units"]) * (model2.1$VCV[,

"traitco:traitco.animal"] + model2.1$VCV[,

"traitco:traitco.units"]))

posterior.mode(genetic.correlation2.3)

HPDinterval(genetic.correlation2.3, 0.95)

#CO:CS, contact object in DL/S/NO vs contact stranger in DL/STR

model2.1 <- MCMCglmm(cbind(cs, co) ~ trait - 1,

random = ~ us(trait):animal,

rcov = ~ us(trait):units,

family = c("gaussian", "gaussian"),

data = pers_lab_mu, nitt = 650000, thin = 1000, burnin = 150000,

prior = prior2, verbose = FALSE)

plot(model2.1$Sol)

autocorr.diag(model2.1$VCV)

#Phenotypic-(within- + among variation) Correlation

genetic.correlation2.3 <- (model2.1$VCV[,

"traitcs:traitco.animal"] + model2.1$VCV[,

"traitcs:traitco.units"]) / sqrt((model2.1$VCV[,

"traitcs:traitcs.animal"] + model2.1$VCV[,

"traitcs:traitcs.units"]) * (model2.1$VCV[,

"traitco:traitco.animal"] + model2.1$VCV[,

"traitco:traitco.units"]))

posterior.mode(genetic.correlation2.3)

HPDinterval(genetic.correlation2.3, 0.95)

#CD:CS, climb down in DL/EP vs contact stranger in DL/STR

model2.1 <- MCMCglmm(cbind(cs, cd) ~ trait - 1,

random = ~ us(trait):animal,

rcov = ~ us(trait):units,

family = c("gaussian", "gaussian"),

data = pers_lab_mu, nitt = 650000, thin = 1000, burnin = 150000,

prior = prior2, verbose = FALSE)

plot(model2.1$Sol)

autocorr.diag(model2.1$VCV)

#Phenotypic-(within- + among variation) Correlation

genetic.correlation2.3 <- (model2.1$VCV[,

"traitcs:traitcd.animal"] + model2.1$VCV[,

"traitcs:traitcd.units"]) / sqrt((model2.1$VCV[,

"traitcs:traitcs.animal"] + model2.1$VCV[,

"traitcs:traitcs.units"]) * (model2.1$VCV[,

"traitcd:traitcd.animal"] + model2.1$VCV[,

"traitcd:traitcd.units"]))

posterior.mode(genetic.correlation2.3)

HPDinterval(genetic.correlation2.3, 0.95)

#(**2)Repeatability**

#Estimation of the coefficients of repeatability, R,

#for each of the three PCs (Table 3 in the main text)

#and trial execution time (ET) based on

#the univariate linear mixed-effect models (LMM)

#for Gaussian distribution with animal ID as a random factor

#using rpt function from the package rptR

#**Table 5 in the main text**

#Repeatability estimates along with their 95% CIs

#for behavioural traits (three (PCs as in Table3, and ET)

#obtained from the univariate LMMs in rptR package

#with animal ID as random factor.

#p- values were estimated with the likelihood ratio test (LRT).

#Data: pers_lab.csv; S1_File.csv in Supporting information files

#should be renamed as pers_lab.csv

pers_lab <- read.csv("D:/Work/R/data/pers_lab.csv",header=TRUE)

pers_lab$trial<- as.factor(pers_lab$trial)

pers_lab$species<- as.factor(pers_lab$species)

pers_lab$animal<- as.factor(pers_lab$animal)

pers_lab$sex<- as.factor(pers_lab$sex)

library(rptR)

#**M.meridianus**

pers_lab_mm <- subset(pers_lab, species == "mm")

#PC1

#Repeatability with no Fixed effects, Boot=1000, no permutations

rep1_boot <- rpt(pc1 ~ (1 | animal), grname = "animal", data = pers_lab_mm, datatype = "Gaussian",

nboot = 1000, npermut = 0)

print(rep1_boot)

#PC2

#Repeatability with no Fixed effects, Boot=1000, no permutations

rep2_boot <- rpt(pc2 ~ (1 | animal), grname = "animal", data = pers_lab_mm, datatype = "Gaussian",

nboot = 1000, npermut = 0)

print(rep2_boot)

#PC3

#Repeatability with no Fixed effects, Boot=1000, no permutations

rep3_boot <- rpt(pc3 ~ (1 | animal), grname = "animal", data = pers_lab_mm, datatype = "Gaussian",

nboot = 1000, npermut = 0)

print(rep3_boot)

#Total Duration

#Repeatability with no Fixed effects, Boot=1000, no permutations

rep4_boot <- rpt(tot_dur ~ (1 | animal), grname = "animal", data = pers_lab_mm, datatype = "Gaussian",

nboot = 1000, npermut = 0)

print(rep4_boot)

#**M.unguiculatus**

pers_lab_mu <- subset(pers_lab, species == "mu")

#PC1

#Repeatability with no Fixed effects, Boot=1000, no permutations

rep1_boot <- rpt(pc1 ~ (1 | animal), grname = "animal", data = pers_lab_mu, datatype = "Gaussian",

nboot = 1000, npermut = 0)

print(rep1_boot)

#PC2

#Repeatability with no Fixed effects, Boot=1000, no permutations

rep2_boot <- rpt(pc2 ~ (1 | animal), grname = "animal", data = pers_lab_mu, datatype = "Gaussian",

nboot = 1000, npermut = 0)

print(rep2_boot)

#PC3

#Repeatability with no Fixed effects, Boot=1000, no permutations

rep3_boot <- rpt(pc3 ~ (1 | animal), grname = "animal", data = pers_lab_mu, datatype = "Gaussian",

nboot = 1000, npermut = 0)

print(rep3_boot)

#Total Duration

#Repeatability with no Fixed effects, Boot=1000, no permutations

rep4_boot <- rpt(tot_dur ~ (1 | animal), grname = "animal", data = pers_lab_mu, datatype = "Gaussian",

nboot = 1000, npermut = 0)

print(rep4_boot)

**#(3) Univariate models for fixed and random (animal ID) effects**

# Four separate univariate Bayesian linear mixed-effect models

#with Markov chain Monte Carlo estimation (MCMCglmm)

#for each of the three PCs (as in Table 3 in the main text)and ET #with species, sex, and the trial number (as a two-level factor)

#included as fixed effects and animal ID as a random factor.

# **Table 7 in the main text**.

#Effects of species, sex, trial number (fixed effects),

#and animal ID (random factor) on behavioural responses

#in gerbils. Posterior estimates along with 95% CRIs derived

#from MCMC linear mixed-effect models run separately for each

#of the three PC and trial execution time – ET.

#Data: pers_lab.csv; S1_File.csv in Supporting information files #should be renamed as pers_lab.csv

#Repeated measures

#Random = animal ID

#95%CI by HPD

pers_lab <- read.csv("D:/Work/R/data/pers_lab.csv",header=TRUE)

pers_lab$trial<- as.factor(pers_lab$trial)

pers_lab$species<- as.factor(pers_lab$species)

pers_lab$animal<- as.factor(pers_lab$animal)

pers_lab$sex<- as.factor(pers_lab$sex)

library(MCMCglmm)

prior1 <- list(G = list(G = list(V = 1e+03, nu = 0.004)), R = list(V = 1, nu = 0.002))

#Model 1 for PC1 For effects of Species + Sex + Trial + #Trial*Species #(pc1 ~ 1 + species + sex + trial + trial*species,

#Random= ~ animal, Gaussian)

model1.1 <- MCMCglmm(pc1 ~ 1 + species + sex + trial + trial*species, random = ~ animal, data = pers_lab, nitt = 650000, thin = 500, burnin = 150000,

prior = prior1, verbose = FALSE)

plot(model1.1$VCV)

autocorr.diag(model1.1$VCV)

#Fixed effects

summary(model1.1)

#Random effect

posterior.mode(model1.1$VCV)

HPDinterval(model1.1$VCV, 0.95)

hist(mcmc(model1.1$VCV)[,"animal"])

#Model 2 for PC2 For effects of Species + Sex + Trial + Trial*Species #(pc2 ~ 1 + species + sex + trial, + trial*species,

#Random= ~ animal, Gaussian)

model1.2 <- MCMCglmm(pc2 ~ 1 + species + sex + trial + trial*species, random = ~ animal, data = pers_lab, nitt = 650000, thin = 500, burnin = 150000, prior = prior1, verbose = FALSE)

plot(model1.2$VCV)

autocorr.diag(model1.2$VCV)

#Fixed effects

summary(model1.2)

#Random effect

posterior.mode(model1.2$VCV)

HPDinterval(model1.2$VCV, 0.95)

hist(mcmc(model1.2$VCV)[,"animal"])

#Model 3 for PC3 For effects of Species + Sex + Trial +Trial*Species

#(pc3 ~ 1 + species + sex + trial + trial*species,

#Random= ~ animal, Gaussian)

model1.3 <- MCMCglmm(pc3 ~ 1 + species + sex + trial + trial*species, random = ~ animal, data = pers_lab, nitt = 650000, thin = 500, burnin = 150000, prior = prior1, verbose = FALSE)

plot(model1.3$VCV)

autocorr.diag(model1.3$VCV)

#Fixed effects

summary(model1.3)

#Random effect

posterior.mode(model1.3$VCV)

HPDinterval(model1.3$VCV, 0.95)

hist(mcmc(model1.3$VCV)[,"animal"])

#Model 4 for Trial Duration For effects of Species + Sex + Trial +

#Trial*Species (tot_dur ~ 1 + species + sex + trial + trial*species,

#Random= ~ animal, Gaussian)

model1.4 <- MCMCglmm(tot_dur ~ 1 + species + sex + trial + trial*species, random = ~ animal, data = pers_lab, nitt = 650000, thin = 500, burnin = 150000, prior = prior1, verbose = FALSE)

plot(model1.4$VCV)

autocorr.diag(model1.4$VCV)

#Fixed effects

summary(model1.4)

#Random effect

posterior.mode(model1.4$VCV)

HPDinterval(model1.4$VCV, 0.95)

hist(mcmc(model1.4$VCV)[,"animal"])
