# Supplementary material for: Multi-assay approach shows species-associated personality patterns in two socially distinct gerbil species
Source: PLoS One. 2024 Apr 16;19(4):e0296214. doi: 10.1371/journal.pone.0296214 (PMC11020386; doi:10.1371/journal.pone.0296214)
Supplement: S1 Table — See Table 1 and Fig 1 for the description of behavioural measurements. (DOCX) [file pone.0296214.s003.docx]

**S1 Table.** Among-individual, within-individual, and phenotypic correlations between behavioural measurements estimated as covariance divided by the square root of sum of variances along with 95% credibility intervals derived from MCMCglmm with animal ID as a random effect. Significant phenotypic correlations are shown in bordered cells and bold underlined font; marginal significant estimates are marked with bold font. See Table 1 for measurements descriptions.

| **Behaviours** | **Among-individual** | | | **Within-individual** | | | **Phenotypic** | | |  | **Among-individual** | | | **Within-individual** | | | **Phenotypic** | | |
| --- | --- | --- | --- | --- | --- | --- | --- | --- | --- | --- | --- | --- | --- | --- | --- | --- | --- | --- | --- |
|  | Estimate | Lower 95CI | Upper 95CI | Estimate | Lower 95CI | Upper 95CI | Estimate | Lower 95CI | Upper 95CI |  | Estimate | Lower 95CI | Upper 95CI | Estimate | Lower 95CI | Upper 95CI | Estimate | Lower 95CI | Upper 95CI |
|  |  |  |  | ***M. meridianus*** | |  |  |  |  |  |  |  |  | ***M. unguiculatus*** | | |  |  |  |
|  |  |  |  |  |  |  |  | **SAME BEHAVIOURS** | | | | |  |  |  |  |  |  |  |
|  |  |  |  |  |  |  |  | **HEAD OUT** | | | | |  |  |  |  |  |  |  |
| **HO_ST:HO_EP** | **0.997** | **0.665** | **0.999** | 0.19 | -0.24 | 0.70 | **0.66** | **0.23** | **0.82** |  | 0.994 | -0.816 | 1.000 | **0.90** | **0.60** | **0.97** | **0.71** | **0.29** | **0.93** |
| **HO_ST:HO_STR** | **0.997** | **0.527** | **0.999** | 0.08 | -0.33 | 0.69 | **0.53** | **0.23** | **0.85** |  | 0.996 | -0.545 | 1.000 | -0.42 | -0.67 | 0.39 | 0.34 | -0.14 | 0.80 |
| **HO_EP:HO_STR** | 0.998 | -0.595 | 1.000 | **0.54** | **0.11** | **0.81** | **0.69** | **0.38** | **0.90** |  | **0.998** | **0.923** | **1.000** | -0.06 | -0.62 | 0.46 | **0.75** | **0.49** | **0.96** |
|  |  |  |  |  |  |  |  | **BODY OUT** | | | | |  |  | |  |  |  |  |
| **BO_ST:BO_EP** | **0.997** | **0.690** | **1.000** | 0.11 | -0.24 | 0.87 | **0.79** | **0.52** | **0.92** |  | 0.989 | -0.939 | 0.999 | 0.46 | -0.25 | 0.77 | **0.51** | **-0.01** | **0.88** |
| **BO_ST:BO_STR** | **0.996** | **0.45** | **0.999** | **0.66** | **0.22** | **0.92** | **0.82** | **0.61** | **0.95** |  | 0.984 | -0.976 | 1.000 | 0.46 | -0.21 | 0.80 | 0.54 | -0.09 | 0.80 |
| **BO_EP:BO_STR** | 0.994 | -0.18 | 1.000 | 0.36 | -0.35 | 0.85 | **0.65** | **0.15** | **0.80** |  | 0.981 | -0.972 | 1.000 | **0.96** | **0.76** | **0.99** | **0.91** | **0.67** | **0.98** |
|  |  |  |  |  |  |  |  | **DIFFERENT BEHAVIOURS** | | | | |  |  |  |  |  |  |  |
| **IMMOB:BO_ST** | 0.991 | -0.151 | 0.999 | -0.17 | -0.70 | 0.49 | 0.42 | -0.08 | 0.81 |  | 0.985 | -0.928 | 0.999 | -0.64 | -0.87 | 0.27 | 0.06 | -0.48 | 0.52 |
| **IMMOB:CO** | 0.990 | -0.726 | 0.999 | -0.17 | -0.52 | 0.42 | 0.14 | -0.21 | 0.59 |  | -0.980 | -1.000 | 0.933 | 0.37 | -0.27 | 0.87 | -0.12 | -0.63 | 0.47 |
| **IMMOB:CD** | 0.982 | -0.873 | 0.998 | -0.01 | -0.73 | 0.60 | 0.24 | -0.30 | 0.63 |  | 0.991 | -0.255 | 1.000 | 0.02 | -0.76 | 0.71 | 0.38 | -0.08 | 0.87 |
| **IMMOB:CS** | **0.995** | **0.672** | **0.999** | -0.14 | -0.58 | 0.47 | **0.51** | **0.14** | **0.82** |  | 0.991 | -0.944 | 1.000 | 0.00 | -0.66 | 0.60 | 0.33 | -0.24 | 0.68 |
| **BO_ST:CO** | **0.996** | **0.117** | **1.000** | -0.17 | -0.44 | 0.57 | **0.46** | **-0.01** | **0.76** |  | 0.977 | -0.982 | 0.997 | **0.56** | **-0.01** | **0.84** | 0.48 | -0.10 | 0.74 |
| **BO_ST:CD** | **0.992** | **0.149** | **1.000** | -0.02 | -0.72 | 0.51 | **0.30** | **0.01** | **0.78** |  | 0.982 | -0.966 | 0.999 | 0.15 | -0.36 | 0.82 | 0.22 | -0.24 | 0.71 |
| **BO_ST:CS** | -0.985 | -0.997 | 0.999 | 0.29 | -0.43 | 0.77 | 0.02 | -0.38 | 0.55 |  | 0.991 | -0.926 | 1.000 | 0.60 | -0.13 | 0.83 | **0.62** | **0.16** | **0.87** |
| **CO:CD** | 0.986 | -0.967 | 0.999 | 0.05 | -0.58 | 0.42 | 0.10 | -0.30 | 0.50 |  | **-0.995** | **-1.000** | **-0.128** | **0.65** | **0.12** | **0.94** | -0.29 | -0.83 | 0.23 |
| **CO:CS** | 0.984 | -0.983 | 0.999 | 0.34 | -0.20 | 0.69 | 0.45 | -0.05 | 0.70 |  | 0.984 | -0.973 | 1.000 | -0.14 | -0.70 | 0.56 | 0.13 | -0.39 | 0.62 |
| **CD:CS** | -0.984 | -0.999 | 0.974 | **0.70** | **0.01** | **0.88** | 0.17 | -0.29 | 0.58 |  | **0.999** | **0.443** | **1.000** | -0.01 | -0.66 | 0.50 | **0.59** | **0.01** | **0.86** |
