# Supplementary material for: Multi-assay approach shows species-associated personality patterns in two socially distinct gerbil species
Source: PLoS One. 2024 Apr 16;19(4):e0296214. doi: 10.1371/journal.pone.0296214 (PMC11020386; doi:10.1371/journal.pone.0296214)
Supplement: Attachment — Submitted filename: Response to Reviewers.docx [file pone.0296214.s004.docx]

**Authors’ Responses (*in italic*) to Editor’s and Reviewers’ Comments**

PONE-D-23-40950

Multi-assay approach shows species-associated personality patterns in two socially distinct gerbil species

**Academic editor’s comments**

After careful consideration, we feel that it has merit but does not fully meet PLOS ONE’s publication criteria as it currently stands. Therefore, we invite you to submit a revised version of the manuscript that addresses the points raised during the review process.

*RE: In the revised MS, we carefully followed PLOS ONE’s publication and style requirements (formatting text, tables, figures, and references, code and data sharing, etc.) and recommendations of the reviewers (see below).*

If you would like to make changes to your financial disclosure, please include your updated statement in your cover letter. Guidelines for resubmitting your figure files are available below the reviewer comments at the end of this letter.

***RE:*** *We included the statement on the funders’ role (“The funders had no role in study design, data collection and analysis, decision to publish, or preparation of the manuscript”) and adjusted figures to PloS One’s standards with the help of PACE as recommended.*

If applicable, we recommend that you deposit your laboratory protocols in [protocols.io](http://protocols.io/) to enhance the reproducibility of your results. Protocols.io assigns your protocol its own identifier (DOI) so that it can be cited independently in the future. For instructions see: <https://journals.plos.org/plosone/s/submission-guidelines#loc-laboratory-protocols>.

***RE:*** *The research protocol is described in detail in the MS, as also was noted by Reviewer #1. So, we don’t think that the depositing protocol is applicable.*

**Journal Requirements:**

When submitting your revision, we need you to address these additional requirements.

1. Please ensure that your manuscript meets PLOS ONE's style requirements, including those for file naming. The PLOS ONE style templates can be found at
2. <https://journals.plos.org/plosone/s/file?id=wjVg/PLOSOne_formatting_sample_main_body.pdf> and
3. <https://journals.plos.org/plosone/s/file?id=ba62/PLOSOne_formatting_sample_title_authors_affiliations.pdf>

*RE: DONE*

2. Please note that PLOS ONE has specific guidelines on code sharing for submissions in which author-generated code underpins the findings in the manuscript. In these cases, all author-generated code must be made available without restrictions upon publication of the work. Please review our guidelines at <https://journals.plos.org/plosone/s/materials-and-software-sharing#loc-sharing-code> and ensure that your code is shared in a way that follows best practice and facilitates reproducibility and reuse.

***RE: DONE.*** *We provided codes as separate Supporting information file (S2_File. docx) along with the data set (S1_File. csv).*

3. Thank you for stating the following financial disclosure:"This research was supported by the Russian Science Foundation (22-14-00223 to AVT, <https://rscf.ru/project/22-14-00223/>)"

Please state what role the funders took in the study.  If the funders had no role, please state: ""The funders had no role in study design, data collection and analysis, decision to publish, or preparation of the manuscript.""

***RE: DONE***

4. Please review your reference list to ensure that it is complete and correct. If you have cited papers that have been retracted, please include the rationale for doing so in the manuscript text, or remove these references and replace them with relevant current references. Any changes to the reference list should be mentioned in the rebuttal letter that accompanies your revised manuscript. If you need to cite a retracted article, indicate the article’s retracted status in the References list and also include a citation and full reference for the retraction notice.

***RE:*** *We omitted one reference from the list as redundant (Schradin, C., Lindholm, A.K., Johannesen, J., Schoepf, I., Yuen, C., Konig, B., & Pillay, N., (2012). Social flexibility and social evolution in mammals: A case study of the African striped mouse (Rhabdomys pumilio ). Molecular Ecology, 21(3), 541–553.)*

**Reviewers' comments:**

Reviewer #1

The existence of personality traits in both species of gerbils is an interesting result, but the main concern of the present research is the small number of individuals included in the study, as also recognized by the authors (rows 674-696). The low numerosity could have affected the results, that shown some inconsistencies related to behavioural syndrome. Although the manuscript is overall interesting, the research could have been partly wrecked by the small dataset. I could suggest to include the part dealing with the issue of the small dataset in the M&M section, and then evaluate any potential effect on the present findings in the Discussion.

***RE: DONE.*** *As recommended, we have moved the passage explaining the “small data set issue” to the M&M section (Lines 138-142) and discuss its possible effects in the Discussion section (Lines 689-707).*

Reviewer #2

While the discussion section is comprehensive, I recommend a careful review to potentially condense its length. Certain parts are challenging to follow, and a more streamlined approach could enhance readability. Additionally, Figure S1 should be incorporated into the main text, as it significantly aids in comprehending the performed behavioral trails.

***RE: DONE*** *as suggested. We shortened the Discussion and omitted redundant and repetitive phrases and passages to condense the discussion and make it more streamlined. We included Figure S1 in the main text (as Fig 1), as recommended.*

In the Materials and Methods section, consider minimizing the use of abbreviations (e.g., DL/S/NO) to improve the narrative flow of the manuscript. Excessive abbreviations can hinder the reader's understanding and disrupt the overall coherence of the text.

***RE: DONE*** *as suggested. We minimized the use of abbreviations throughout the text.*

Reviewer's Responses to Questions

Comments to the Author

1. Is the manuscript technically sound, and do the data support the conclusions?

The manuscript must describe a technically sound piece of scientific research with data that supports the conclusions. Experiments must have been conducted rigorously, with appropriate controls, replication, and sample sizes. The conclusions must be drawn appropriately based on the data presented.

Reviewer #1: Partly

Reviewer #2: Yes

2. Has the statistical analysis been performed appropriately and rigorously?

Reviewer #1: Yes

Reviewer #2: Yes

3. Have the authors made all data underlying the findings in their manuscript fully available?

The [PLOS Data policy](http://www.plosone.org/static/policies.action#sharing) requires authors to make all data underlying the findings described in their manuscript fully available without restriction, with rare exception (please refer to the Data Availability Statement in the manuscript PDF file). The data should be provided as part of the manuscript or its supporting information, or deposited to a public repository. For example, in addition to summary statistics, the data points behind means, medians and variance measures should be available. If there are restrictions on publicly sharing data—e.g. participant privacy or use of data from a third party—those must be specified.

***RE: DONE.*** *We provided the dataset along with codes as separate Supporting information files (S1_File.csv and S2_File.docx, respectively)*

Reviewer #1: Yes

Reviewer #2: Yes

4. Is the manuscript presented in an intelligible fashion and written in standard English?

PLOS ONE does not copyedit accepted manuscripts, so the language in submitted articles must be clear, correct, and unambiguous. Any typographical or grammatical errors should be corrected at revision, so please note any specific errors here.

Reviewer #1: Yes

Reviewer #2: Yes

5. Review Comments to the Author

Reviewer #1:

In the Introduction the personality and behavioural sindrome were thoroughly discussed and the cited references were more than enoughto define the experimental problem adreesed by the research.

Although the dataset is quite small (12 and 9 individuals, respectively) the experimental procedure was correct, and described in details allowing thus the repeatibility of the experiment. Each survey was perfomed twice, at a 3-days interval, as suggested by literature. The M&M part is clear and the protocol is well-described. The chosen statistical analyses are sound and well-supported by the references.

The results are clearly shown, and no further analyses are required. In the Discussion the findings are assessed, and supported by the references. The alternative hypothesis suggested for the results contradicting the behavioural sindrome concept is acceptable.

The existence of personality traits in both species of gerbils is an interesting result, but the main concern of the present research is the small number of individuals included in the study, as also recognized by the authors (rows 674-696). The low numerosity could have affected the results, that shown some inconsistencies related to behavioural syndrome. Although the manuscript is overall interesting, the research could have been partly wrecked by the small dataset. I could suggest to include the part dealing with the issue of the small dataset in the M&M section, and then evaluate any potential effect on the present findings in the Discussion.

***RE: DONE.*** *As recommended, we moved the passage explaining the “small data set issue” to the M&M section (****Lines 138-142****) and discuss its possible effects in the Discussion section (****Lines 689-707****).*

Reviewer #2:

Having thoroughly reviewed the manuscript titled "Multi-assay approach shows species-associated personality patterns in two socially distinct gerbil species," I find no significant methodological difficulties or other issues that require immediate attention.

The articulation of objectives appears accurate and aligns well with the introduction, methods, and results. The interpretation of the findings not only prompts further investigation but also encourages a more in-depth analysis of various aspects related to animal personality and behavioral syndromes in small mammals, particularly rodents. To substantiate the social niche specialization hypothesis, additional studies are warranted, delving into the mechanisms of phenotypic integration and unraveling the evolutionary paths leading to the development of behavioral syndromes.

While the discussion section is comprehensive, I recommend a careful review to potentially condense its length. Certain parts are challenging to follow, and a more streamlined approach could enhance readability. Additionally, Figure S1 should be incorporated into the main text, as it significantly aids in comprehending the performed behavioral trails.

***RE: DONE*** *as recommended (see above).*

In the Materials and Methods section, consider minimizing the use of abbreviations (e.g., DL/S/NO) to improve the narrative flow of the manuscript. Excessive abbreviations can hinder the reader's understanding and disrupt the overall coherence of the text.

***RE: DONE*** *as recommended (see above).*

Overall, I believe this manuscript constitutes a valuable contribution to the field of animal personality and behavioral syndromes in non-model small mammal species.

6. PLOS authors have the option to publish the peer review history of their article ([what does this mean?](https://journals.plos.org/plosone/s/editorial-and-peer-review-process#loc-peer-review-history)). If published, this will include your full peer review and any attached files.

If you choose “no”, your identity will remain anonymous but your review may still be made public.

**Do you want your identity to be public for this peer review?** For information about this choice, including consent withdrawal, please see our [Privacy Policy](https://www.plos.org/privacy-policy).

Reviewer #1: No

Reviewer #2: No
